# Prevalence and factors associated with a prescription of a Lyme borreliosis serology for erythema migrans diagnosis in general practice: a study from the French sentinel network, 2009–2020

**DOI:** 10.1186/s12875-023-02108-3

**Published:** 2023-08-24

**Authors:** Camille Bonnet, Julie Figoni, Cécile Souty, Alexandra Septfons, Sylvie de Martino, Henriette de Valk, Lucie Fournier, Thomas Hanslik, Benoît Jaulhac, Thierry Blanchon

**Affiliations:** 1 Sorbonne Université, INSERM, Institut Pierre Louis d’Épidémiologie et de Santé Publique, IPLESP, Paris, F75012 France; 2 Santé publique France, Saint-Maurice, F94410 France; 3 CNR des Borrelia et Institut de Bactériologie, Fédération de Médecine Translationnelle de Strasbourg, University of Strasbourg, CHRU Strasbourg, UR7290, ITI InnoVec, Strasbourg, 67000 France; 4 Service de Médecine Interne, APHP, Hôpital Ambroise Paré, Assistance Publique - Hôpitaux de Paris, Boulogne-Billancourt, France; 5 Université Versailles Saint-Quentin-en-Yvelines, UVSQ, UFR de Médecine Simone Veil, Versailles, France

**Keywords:** Lyme borreliosis, Erythema Chronicum Migrans, Serologic tests, General Practice, Sentinel surveillance

## Abstract

**Background:**

Serological testing of patients consulting for typical erythema migrans (EM) is not recommended in European recommendations for diagnosis of Lyme borreliosis (LB). Little is known on the level of adherence of French general practitioners to these recommendations. The objectives were to estimate the proportion of Lyme borreliosis serological test prescription in patients with erythema migrans seen in general practice consultations in France, and to study the factors associated with this prescription.

**Methods:**

LB cases with an EM reported by the French general practitioners (GPs) of the *Sentinelles network* between January 2009 and December 2020 were included. To assess the associations with a prescription of a serological test, multilevel logistic regression models were used.

**Results:**

Among the 1,831 EM cases included, a prescription for a LB serological test was requested in 24.0% of cases. This proportion decreased significantly over the study period, from 46.8% in 2009 to 15.8% in 2020. A LB serological prescription was associated with patients with no reported tick bite (Odds Ratio (OR): 1.95; 95% confidence interval [1.23–3.09]), multiple EM (OR: 3.82 [1.63–8.92]), EM of five centimeters or more (OR: 4.34 [2.33–8.08]), and GPs having diagnosed less than one EM case per year during the study period (OR: 5.28 [1.73–16.11]).

**Conclusions:**

Serological testing of patients consulting for EM is not recommended in European recommendations for diagnosis of Lyme borreliosis. Therefore, the significant decrease in the rate of LB serological test for EM over the study period is encouraging. The factors identified in this study can be used to improve messaging to GPs and patients. Further efforts are needed to continue to disseminate diagnostic recommendations for LB to GPs, especially those who rarely see patients with EM.

## Introduction

Lyme borreliosis (LB) is the most prevalent tick-borne infection in Europe and in North America [1]. LB is caused by the transmission of *Borrelia burgdorferi* sensu lato spirochetes hosted by *Ixodes ricinus* ticks [2]. Erythema migrans (EM) is the most common early clinical manifestation of the infection [3]. Its typical form is defined by a characteristic circular, sharp edged skin lesion, red with central clearing (bull’s eye or target lesion) with a diameter of five centimeters or more, expanding in a centrifugal growth and usually without pruritus. It occurs several days to weeks after a tick bite and may be associated with non-specific clinical signs such as headache, fever, myalgia and fatigue [4, 5]. Without treatment, progression of the disease may lead to more severe forms in a small proportion of patients.

In France, the incidence rate of Lyme borreliosis is estimated at 91 cases per 100,000 in 2020 [95% confidence interval; 80–102], with wide variations between regions [6]. EM usually represents more than 95% of LB cases seen in primary care [7]. The presence of a typical EM is sufficient to confirm the diagnosis of LB [4, 8]. In this early stage of infection, serological confirmation is not recommended in France and in most countries who had published diagnostic guidelines, except in very specific conditions, since a false negative test may occur and the specificity of the clinical presentation is mostly sufficient to treat the patient [9, 10]. Furthermore, a positive test at this stage can reflect a previous infection, especially in individuals with repeated exposures [11]. The European society of clinical microbiology and infectious disease (ESCMID) study group for Lyme borreliosis (ESGBOR) recommends since 2011, to not prescribe serological test in case of typical EM [12, 13]. The French recommendations are in line with these European guidelines and have not changed since 2006 [14–16].

Little is known about the level of adherence of French general practitioners (GP) to the guidelines regarding the non-prescription of a LB serological test for patients presenting an EM. In a study conducted between 1999 and 2000 in France, it was reported that LB serology was performed in 45% of patients with EM [17]. Other studies in Europe showed that practices of prescribing serological LB test were more often not in line with the national guidelines [18–20]. Therefore, we aimed to estimate the proportion of LB serological test prescription in patients with an EM seen in general practice in France and to determine the factors associated with this prescription.

## Method

### Data sources

This study used data from the *Sentinelles network*, which is a real-time surveillance system since 1984 in primary care in France, developed in collaboration with the French public health agency (Santé publique France) and the national microbiological reference laboratory. General practitioners of the *Sentinelles network* (SGPs) are spread throughout France and their participation is voluntary and unpaid. They constitute a representative sample of the national GPs in terms of age and type of activity [21]. Since 2009, they report new LB cases seen in consultation on a weekly basis.

### Data collected and study period

*Sentinelles network* uses case definition proposed by the study group for Lyme borreliosis (ESGBOR) [12], which includes EM. Cases with suspected EM less than five centimeters in size are included in the definition, except when a tick bite and a delay of less than two days between the date of the bite and the date of diagnosis are mentioned. For each LB case reported by a SGP, data are collected from a standardized questionnaire to describe the clinical manifestations, including the presence of an erythema migrans, its size (in centimeters) and expanding nature, aspect, number of lesions, presence of other clinical manifestations, blood serological test prescription for this episode as well as the history of tick bites preceding the symptoms. Basic characteristics of the patients such as age and sex are also collected. A validation of each case was performed by an expert group constituted by clinicians, microbiologists and epidemiologists.

Our study population includes all validated LB cases with an erythema migrans seen in consultation by a SGP between January 2009 and December 2020.

### Measures

Our primary outcome was the prescription of a LB serological test for the episode of EM for which the patient came to consult in general practice. To assess the association between the incidence of LB in the SGPs work location region and prescription of LB serological tests, we built a three-category variable depending on the mean LB incidence rate by region over the period. A mean regional LB incidence rate ≥ 100 cases per 100 000 population was categorized as “high incidence rate”, “moderate incidence rate” for regions with an incidence between 50 and 99 LB cases per 100 000 population, and “low incidence rate” for incidence rates of less than 50 LB cases per 100 000 population. To determine if there was an association between SGPs experience with LB and prescription practices, we defined an indicator using the frequency of LB cases seen in consultation by SGPs per year over the entire period, taking into account the number of weeks of active participation to the *Sentinelles network*.

### Analysis

We described the LB cases and characteristics of the SGPs who reported the cases. The proportion of EM episodes for which a LB serology was prescribed was calculated by year over the study period and the trend was evaluated with Cochran-Armitage tests. To assess the association between each factor and serological test prescription, we subsequently conducted a descriptive analysis and a series of bivariate multilevel logistic regression models. Multilevel models were used to take into account the hierarchical structure of our data (episodes within SGPs) and the non-independence of observations within SGPs. In our final model, adjusted odds ratios and their 95% confidence intervals were estimated using a multivariable multilevel logistic regression model. Since information on EM size and its expanding nature was not collected in 2009 and 2010, the analysis of factors associated with the prescription of a LB serological test has been limited to cases reported from 2011 onwards. All statistical analyses were performed using R Software.

## Results

Over an 11-year period, 1,831 cases of LB presenting an EM were reported by 427 SGPs. The characteristics of patients, episodes and SGP are presented in Table 1. About 31.9% of the patients were between 50 and 65 years-old and 10.1% were 15 years-old or less. Women represented 53.6% of the cases (n = 957). Almost all EM were single EM (95.3%, n = 1,706) and most (77.5%, n = 1,282) were five centimeters or more. For 72.8% of the cases (n = 1,176), a tick bite was reported.

**Table 1:** Description of EM cases characteristics (patients and GPs who declared the case) N = 1831

	N (%)
*Patient characteristics*	
**Age, in years (m.d.=5)**	
≤ 15	185 (10.1)
]15–35]	289 (15.8)
]35–50]	364 (19.9)
]50–65]	583 (31.9)
More than 65	405 (22.2)
**Gender (m.d.=45)**	
Female	957 (53.6)
Male	829 (46.4)
**Reported tick bite (m.d.=215)**	
Yes	1176 (72.8)
No	440 (27.2)
**Erythema migrans size** ^**1**^ **(m.d.=176)**	
< 5 cm	373 (22.5)
≥ 5 cm	1282 (77.5)
**Type of EM (m.d.=40)**	
Single	1706 (95.3)
Multiple	85 (4.7)
**Centrifugal growth/expansion** ^**1**^ **(m.d.=338)**	
Yes	1403 (94.0)
No	90 (6.0)
**Prescription of a serology (m.d.=88)**	
Yes	418 (24.0)
No	1325 (76.0)
*SGPs characteristics*	
**Age, in years (m.d.=17)**	
≤ 40	344 (19.0)
]40–50]	427 (23.5)
]50–60]	593 (32.7)
> 60	450 (24.8)
**Gender (m.d.=0)**	
Female	553 (30.2)
Male	1278 (69.8)
**Type of practice (m.d.=131)**	
Individual	678 (41.5)
In groups	957 (58.5)
**Regions by incidence rates** ^**2,3**^ **(m.d.=0)**	
High incidence region	864 (47.2)
Moderate incidence region	374 (20.4)
Low incidence region	593 (32.4)
**Type of municipality of practice (m.d.=2)**	
Rural	540 (29.5)
Urban	1289 (70.5)
**Number of cases seen by SGPs on a yearly basis** **(m.d.=0)**	
≤ 1 case	490 (26.8)
]1–5[	943 (51.5)
≥ 5 cases	398 (21.7)
**Study period (m.d.=0)**	
2009–2014	498 (27.2)
2015–2020	1333 (72.8)

m.d. = Missing data

^1^Information not collected in 2009 and 2010

^2^Regions of SGP’s practice

^3^Low incidence rate: < 50 cases/100 000 ; moderate incidence rate: [50–100[ cases/100 000 ; high incidence rate: ≥ 100 cases/100 000

**Fig. 1:**
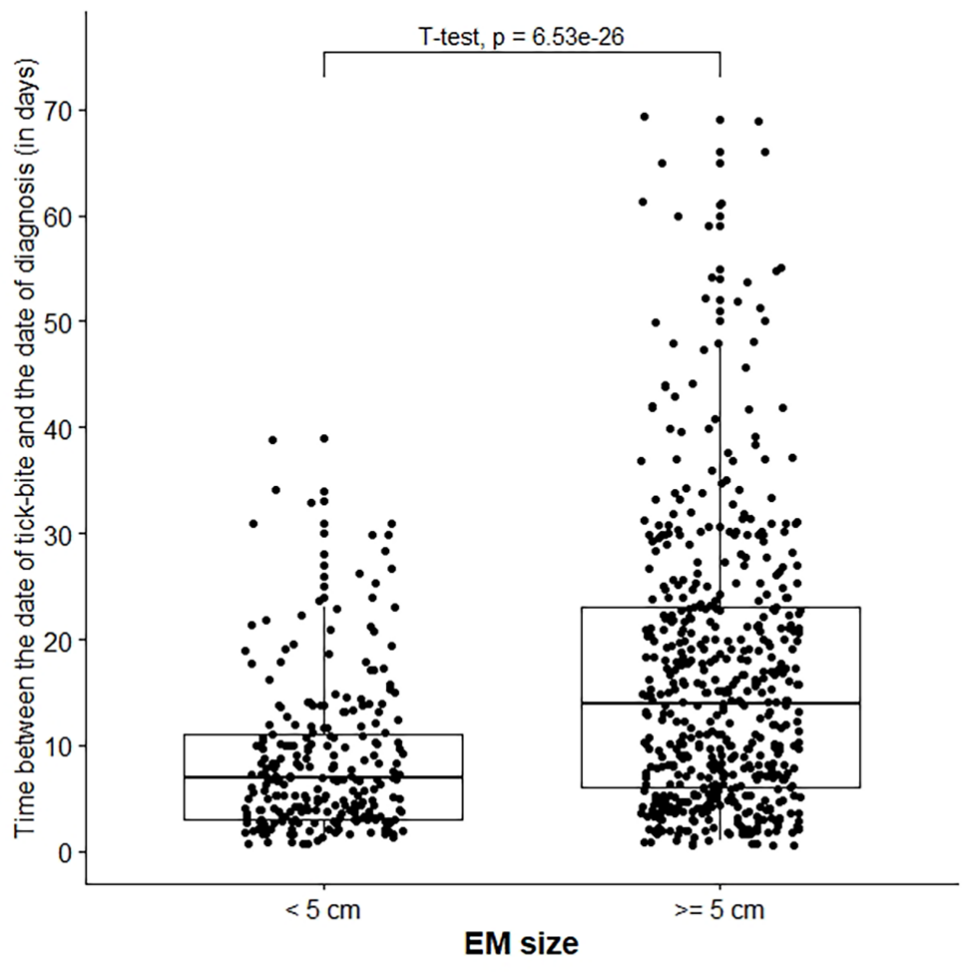
Distribution of time between tick bite and GP diagnosis date by erythema migrans size, N = 791

As expected, the median time between the tick bite and the date of consultation with the SGP was significantly shorter in patients with EM less than five centimeters than those with EM of five centimeters or more (7 days vs. 14 days; p-value < 0.001) (Fig. 1). Almost half of the cases (47.2%, n = 864) were reported by SGPs practicing in a high LB incidence region. About 26.8% of cases (n = 490) were reported by SGPs having seen one LB case or less per year in consultation, and 21.7% (n = 398) were reported by SGPs having seen five cases or more per year.

**Fig. 2:**
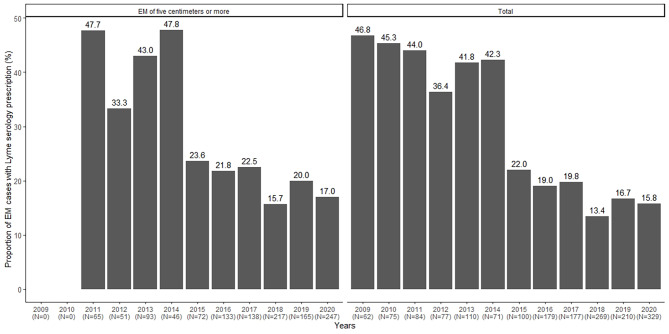
Proportion of Lyme borreliosis serological test prescriptions in patients with erythema migrans of five centimeters or greater (N = 1,227) (*on the left*) and overall analysis population (N = 1,743) (*on the right*)

Overall 24.0% (n = 418) of EM cases had a LB serological test prescription (Fig. 2). Serology prescription status was unknown for 88 cases (4.8%). The percentage of cases with a LB serology prescription decreased steadily over the period from 46.8% to 2009 to 15.8% in 2020 (*p-value < 0.0001*). In patients with an EM of five centimeters or more, this proportion was 24.1% over the entire period and reached 17.0% in 2020 (*p-value < 0.0001*).

Table 2 shows the proportion of requested serological tests according to episodes’ and SGPs’ characteristics and the results of uni- and multivariable analyses. In multivariate analysis patients without a reported tick bite (aOR = 1.95 [1.23–3.09]), with an EM of five centimeters or more (aOR = 4.34 [2.33–8.08]) or with multiple EM (aOR = 3.82 [1.63–8.92]) were more likely to be prescribed a LB serological test. SGPs reporting one case or less per year were more likely to prescribe a LB serological test in case of EM than SGPs reporting five cases or more each year (aOR = 5.28 [1.73–16.11]). The factors identified were similar in the sensitivity analysis conducted in subgroups in patients with EM of five centimeters or more (not shown).

**Table 2:** Characteristics associated with serological prescription among EM episodes reported between 2011 and 2020, N = 1684

	Prescription of a serological test				
	n in row (%)	Crude OR [95% CI]	*p-value*	Adjusted OR [95% CI]	*p-value*
*Patients and episodes characteristics*					
**Age, in years**			*0.21*		*0.94*
≤ 15	26 (16.6)	0.70 [0.37–1.32]		0.83 [0.37–1.87]	
]15–35]	56 (23.1)	1.06 [0.63–1.79]		0.94 [0.46–1.89]	
]35–50]	70 (21.7)	Ref.		Ref.	
]50–65]	127 (24.5)	1.36 [0.88–2.10]		1.11 [0.63–1.96]	
More than 65	76 (20.9)	1.01 [0.63–1.63]		0.95 [0.51–1.77]	
**Gender**			*0.16*		*0.15*
Female	192 (23.2)	Ref.		Ref.	
Male	155 (21.1)	0.80 [0.58–1.09]		0.74 [0.49–1.11]	
**Reported tick bite**			*< 0.001*		*< 0.01*
Yes	196 (19.0)	Ref.		Ref.	
No	108 (27.1)	2.21 [1.52–3.20]		1.95 [1.23–3.09]	
**Erythema migrans size**			*< 0.001*		*< 0.001*
< 5 centimeters	49 (13.9)	Ref.		Ref.	
≥ 5 centimeters	296 (24.1)	3.04 [1.94–4.74]		4.34 [2.33–8.08]	
**Type of EM**			*< 0.001*		*< 0.01*
Single	319 (21.0)	Ref.		Ref.	
Multiple	30 (42.2)	3.73 [1.91–7.29]		3.82 [1.63–8.92]	
**Centrifugal growth**			*0.32*		*0.42*
Yes	278 (20.5)	Ref.		Ref.	
No	21 (25.0)	1.43 [0.71–2.86]		1.41 [0.61–3.26]	
*SGPs characteristics*					
**Age, in years**			*0.06*		*0.37*
≤ 40	58 (17.7)	Ref.		Ref.	
]40–50]	90 (24.1)	1.95 [1.02–3.70]		1.61 [0.77–3.35]	
]50–60]	123 (25.1)	2.39 [1.26–4.52]		0.94 [0.43–2.05]	
More than 60	76 (18.9)	1.79 [0.90–3.55]		1.32 [0.56–3.11]	
**Gender**			*0.18*		*0.55*
Female	111 (22.4)	Ref.		Ref.	
Male	244 (22.0)	1.45 [0.84–2.52]		0.83 [0.44–1.56]	
**Type of practice**			*0.09*		*0.72*
Individual	136 (23.7)	Ref.		Ref.	
In groups	180 (20.7)	0.60 [0.33–1.08]		0.89 [0.48–1.66]	
**Municipality of practice**			0.60		*0.85*
Rural	97 (20.9)	Réf.		Réf.	
Urban	258 (22.6)	1.16 [0.66–2.05]		1.06 [0.56–2.03]	
**Regions by incidence rates** ^**1,2**^			*0.52*		*0.16*
Low incidence region	139 (27.0)	1.37 [0.77–2.43]		1.93 [0.94–3.96]	
Moderate incidence region	74 (23.8)	1.34 [0.67–2.70]		1.84 [0.81–4.18]	
High incidence region	141 (18.2)	Ref.		Ref.	
**Number of cases seen by SGPs on a yearly basis**			*< 0.001*		*< 0.01*
≤ 1 case	150 (38.5)	5.42 [2.48–11.84]		5.28 [1.73–16.11]	
]1–5[	168 (20.6)	2.18 [1.00–4.76]		2.19 [0.77–6.20]	
≥ 5 cases	37 (9.3)	Ref.		Ref.	
**Year of diagnosis**			*< 0.001*		*< 0.001*
2011–2014	141 (41.2)	4.79 [3.21–7.16]		4.80 [2.77–8.33]	
2015–2019	214 (16.9)	Ref.		Ref.	

Adjusted odds ratio for all variables in the table; Ref.: reference.

^1^Regions of SGP’s practice

^2^Low incidence rate: < 50 cases/100 000 ; moderate incidence rate: [50–100[ cases/100 000 ; high incidence rate: ≥ 100 cases/100 000

## Discussion

This study provides information on prescribing practices of GPs of LB serological tests in cases of EM over a 12-year period and factors associated with this prescription over the ten last years in France. As explained above, antibody testing is not recommended, by consensus in most of the European countries, for the diagnosis of LB in patients with EM.

This proportion has strongly decreased over the period. Our results show a considerable improvement in awareness and adherence to the French guidelines for testing in patients with EM in our country between 2009 and 2020, with a marked improvement from 2015 onwards. One hypothesis to explain this clear decrease from 2015 may be the publication of an expert report on LB by the French Public Health Council in 2014, which may have led to an increased dissemination of recommendations to GPs [22]. The French Infectious Diseases Society, the French Society of Dermatology and French National Reference Center for *Borrelia* have also participated in the dissemination of these recommendations, particularly through continuing medical education for physicians and biologists. Although the interpretation of cross-country comparisons is limited due to the heterogeneity of contexts, several studies in other European countries and in Canada have reported excessive serological testing or lack of adherence to national recommendations for patients with EM seen in general practice [18, 19, 23–26]. In a study conducted in the Netherlands between 2010 and 2015 on the diagnostic behavior of GPs towards LB, Botman et al. found that a serology was prescribed for 18% of patients with typical EM [25]. Vanthomme et al. used data from the Belgian sentinel network to study the management of LB suspicion in 2003–2004 and 2008–2009. They showed that half of the patients with EM were tested serologically and no improvement was observed between the two periods [27]. Finally, in a retrospective study in Norway between 2005 and 2009, 15% of patients with EM episodes were tested serologically [24].

Our results also showed that a LB serological test was more frequently requested for the less typical forms of EM (without reported tick-bite or multiple EM). In a recent qualitative study conducted with sixteen German GPs, they described that some GPs may order a LB serological test because “they want to be sure” without waiting for the result to treat [28]. In contrast, the significant association with EM size is less expected. One hypothesis to explain this result could be that GPs are prescribing serology more frequently for EM of five centimeters or more since the risk of a false negative result decreases over time, as the size of the EM is increasing. The serological test in this situation is not necessary but can be reassuring for the GPs, even if a positive result only shows previous exposure to *B. burgdorferi*. Another hypothesis is that the notion of a tick bite is less frequently reported in patients with EM of five centimeters or more, compared to those with EM of less than five centimeters (66% *versus* 88% ; p-value < 0.001, results not shown). The absence of a tick bite can lead to doubts and confusion on the part of the GPs and that encourages patient-GPs to confirm the diagnosis of LB by serology. These hypothesis is supported by the finding that GPs rarely seeing Lyme cases in consultation were more likely to prescribe a LB serology to a patient presenting a suspicion of EM, although no significant difference was found between regions. It is likely that GPs who see more frequent Lyme cases have better knowledge of the disease and its management and therefore their practices are more in line with the most recent guidelines. In addition, there may be differences in training offer and knowledge sharing at more local levels. The absence of association with the region of practice can be explained by the fact that a high heterogeneity of knowledge and practices may exist between SGPs practicing within the same region. These results are in line with Botman et al. who reported wide disparities between the practices of the twelve GPs participating in the study [25].

This study has several limitations. The knowledge of GPs about LB in our study could be better than that of other French GPs due to their special interest in public health and their participation to the *Sentinelles network*. Unfortunately, we do not have data on GP knowledge of guidelines and recommendations regarding LB serology testing. We have made the hypothesis that LB serology was prescribed by GPs seeing the case in consultation, but we have no certainty about the healthcare professional who prescribed it. The patient might have visited another GP before or another practitioner who did the prescription, but we assume such situations to be rare. We do not have information on the context of the prescription or reasons that could have motivated the GP to prescribe a serology, nor on patient’s follow-up or the various tests performed. In some cases with a non-typical presentation, some GPs could have asked for a serology to observe a seroconversion. Because of the context surrounding LB in France, we can assume that in some cases, the GP prescribed the LB serology because of the patient’s expectations and demands [29]. The main strengths of this study are the national coverage and the large sample of cases, included using a common protocol, and validated by an expert group. It is therefore possible to compare prescription practices over the study period. Participating GPs are representative of GPs in France in terms of age and type of activity.

In conclusion, GPs’ adherence to recommendations regarding the prescription of LB serology in patients with erythema migrans has improved significantly over the past 12 years. These results are encouraging to continue and focus actions. It is essential to make the recommendations more accessible to GPs who are rarely seeing patients with LB. Studies are still needed to better understand the difficulties that GPs are facing in managing LB according to the recommendations and to identify the barriers to implement these recommendations.

## Data Availability

The datasets used and/or analysed during the current study available from the corresponding author on reasonable request.

## List of abbreviations

aOR: Adjusted odds ratio
CI: Confidence interval
EM: Erythema migrans
GP: General practitioners
LB: Lyme borreliosis
OR: Odds ratio
SGP: Sentinel general practitioners
